# Use of Network Analysis and Spread Models to Target Control Actions for Bovine Tuberculosis in a State from Brazil

**DOI:** 10.3390/microorganisms9020227

**Published:** 2021-01-22

**Authors:** Nicolas Cespedes Cardenas, Pilar Pozo, Francisco Paulo Nunes Lopes, José H. H. Grisi-Filho, Julio Alvarez

**Affiliations:** 1 Department of Preventive Veterinary Medicine and Animal Health, School of Veterinary Medicine and Animal Science, University of São Paulo, São Paulo 05508, Brazil; ncespedesc@unal.edu.co (N.C.C.); jgrisi@usp.br (J.H.H.G.-F.); 2 VISAVET Health Surveillance Centre, Universidad Complutense de Madrid, 28040 Madrid, Spain; ppozo@ucm.es; 3 MAEVA SERVET S.L. Alameda del Valle, 28749 Madrid, Spain; 4 Secretary of Agriculture, Livestock and Agribusiness of State of Rio Grande do Sul (SEAPA-RS), Porto Alegre 95150-900, Brazil; francisco-lopes@seapdr.rs.gov.br; 5 Departamento de Sanidad Animal, Facultad de Veterinaria, Universidad Complutense de Madrid, 28040 Madrid, Spain

**Keywords:** bovine tuberculosis, network analysis, disease modeling, epidemiology, mycobacterium bovis, cattle, control disease

## Abstract

Livestock movements create complex dynamic interactions among premises that can be represented, interpreted, and used for epidemiological purposes. These movements are a very important part of the production chain but may also contribute to the spread of infectious diseases through the transfer of infected animals over large distances. Social network analysis (SNA) can be used to characterize cattle trade patterns and to identify highly connected premises that may act as hubs in the movement network, which could be subjected to targeted control measures in order to reduce the transmission of communicable diseases such as bovine tuberculosis (TB). Here, we analyzed data on cattle movement and slaughterhouse surveillance for detection of TB-like lesions (TLL) over the 2016–2018 period in the state of Rio Grande do Sul (RS) in Brazil with the following aims: (i) to characterize cattle trade describing the static full, yearly, and monthly snapshots of the network contact trade, (ii) to identify clusters in the space and contact networks of premises from which animals with TLL originated, and (iii) to evaluate the potential of targeted control actions to decrease TB spread in the cattle population of RS using a stochastic metapopulation disease transmission model that simulated within-farm and between-farm disease spread. We found heterogeneous densities of premises and animals in the study area. The analysis of the contact network revealed a highly connected (~94%) trade network, with strong temporal trends, especially for May and November. The TLL cases were significantly clustered in space and in the contact network, suggesting the potential for both local (e.g., fence-to-fence) and movement-mediated TB transmission. According to the disease spread model, removing the top 7% connected farms based on degree and betweenness could reduce the total number of infected farms over three years by >50%. In conclusion, the characterization of the cattle network suggests that highly connected farms may play a role in TB dissemination, although being close to infected farms was also identified as a risk factor for having animals with TLL. Surveillance and control actions based on degree and betweenness could be useful to break the transmission cycle between premises in RS.

## 1. Introduction

Livestock movements create complex dynamic interactions among premises that can be represented, interpreted, and used for epidemiological purposes [1,2,3]. These movements are a very important part of the production chain but may also contribute to the spread of infectious diseases through the transfers of infected animals within premises over large distances [4,5].

Movements between premises are not distributed homogeneously. Instead, some premises usually play a key role in the trade flow and could therefore have a higher risk of being exposed to infectious diseases and of contributing to their spread [4,6]. For this reason, the identification of these premises can be a very useful tool for disease control and eradication programs in order to help in minimizing disease transmission. One of the diseases subjected to control programs in many countries around the world is bovine tuberculosis (TB), and specifically in Brazil is the focus of the National Control and Eradication Program of Bovine Tuberculosis (PNCEBT) [7].

This program aims to reduce the incidence and prevalence of bovine tuberculosis (TB) by performing intradermal tuberculin tests, culling positive animals, and detecting TB-like lesions (TLL) in slaughterhouses as well as monitoring and controlling the transit of animals between different regions. In Brazil, the first program was launched in 2001 and was reviewed in 2016, when the epidemiological status of 13 states was described showing a heterogeneous herd prevalence among and within regions that ranged between zero and 13.9% [7,8].

In recent years, the availability of a high number of records and high-quality data has helped to evaluate and improve animal disease surveillance programs in general (and bovine tuberculosis programs in particular) worldwide [9,10]. In this sense, social network analysis (SNA) tools have been used to characterize cattle trade patterns and to identify highly connected premises that may act as hubs in the movement network. This can be useful to implement targeted control measures in order to reduce the incidence of communicable diseases such as TB, and especially in low-prevalence regions [11,12]. However, the potential of this approach has not been assessed in Brazil yet. In this context, the Brazilian official veterinary service in particular has the necessary resources to approach the management of infectious diseases more efficiently using powerful tools like SNA [13,14].

This study focusses on cattle trade from Rio Grande do Sul state (RS), Brazil, with a reported bovine population in 2018 of 12,451,432 animals, which represents the sixth largest population in the country and the largest in the southern region [15]. Most of the farms have a closed cycle (from breeding to fattening) and beef production is the predominant activity, followed by a mix of beef production and dairy production, and then sole dairy production. In the last TB report in 2016, this state reported a herd prevalence of 2.8% (95% confidence interval (CI): 1.8, 4.0), and animal prevalence of 0.7% (95% CI 0.4, 1) [16].

The main control strategy for TB in RS is based on post-mortem inspection at the slaughterhouse, where a visual and manual examination of the carcass is conducted along with incisions of a defined range of tissues subjected to further visual examination in order to detect possible TLL. When a TLL is detected in the slaughterhouse, the movement of animals from the premises of origin is temporarily restricted, except for movements to slaughterhouses, until it regains its TB-free status by testing negative in the intradermal test, among others. Additionally, all farms that ship animals for breeding purposes must have a negative intradermal test regardless of farm status [17].

In this region, active surveillance using the intradermal tuberculin test is not mandatory, and this test is generally applied for voluntary certification of free breeding establishments for brucellosis and tuberculosis in the frame of the PNCEBT program. Therefore, the detection of TLL plays an important role in the detection and control of TB in the population [17].

Risk-based surveillance focuses on the subset of the population with a higher risk of infection; thus, the sensitivity of the surveillance system reduces funding and labor costs. Hence, the modeling of TB through the space and trade network can help to design risk-based surveillance activities, especially in the case of TB, since a primary way of spread is the introduction of infected cattle through cattle movements [6,12,18,19].

Transmission models based on network movements allow simulation of disease spread and can therefore be useful for the evaluation of control measures targeted to specific highly connected premises to ultimately estimate the transmission chain and the potential number of affected herds [20]. However, network-based modeling approaches for TB are challenging in part due to the long latent periods, low within-premises transmission rates, and limitations of diagnostic tests [12,21].

Therefore, to accurately estimate between-premises spread of TB, transmission models must run over long time periods and incorporate within-premises dynamics, including changes in within-premises prevalence over time to provide comprehensive quantitative representations of disease transmission pathways [12,22].

The aims of this study were: (i) to characterize the cattle trade in RS state in Brazil from 2016 to 2018 describing the static full, yearly, and monthly snapshots of the network contact trade, (ii) to identify clusters of TLL-positive premises in the spatial and movement networks as a proxy of the likelihood of spatial or movement-mediated TB transmission, and (iii) to evaluate the potential of TB spread and targeted control actions using a Susceptible-Occult-Reactive-Infectious network model to inform the risk-based TB surveillance.

## 2. Materials and Methods

### 2.1. Datasets Description

All movements of animals between premises are mandatorily reported to the official veterinary service to obtain a certificate that allows the transport of animals in RS, Brazil. We used this data provided by the official veterinary service of the state. The database included all cattle movements with origin and destination in premises located in RS. For each movement, the following information was available: origin and destination (IDs and longitude and latitude coordinates), date, and purpose of the movement (see below).

The whole dataset included 1,482,920 movement records. Incomplete or incorrect data (movements with missing IDs, same origin and destination, duplicates) (*n* = 8532) were removed prior to the analysis so that the final database included 1,474,388 movements. For the static, temporal network, and k-test analysis (see below), movements to slaughterhouses (36.54%, *n* = 538,804) were excluded (only for the network analysis and description) as these were assumed to be epidemiological endpoints.

The births dataset included 733,008 records (premises ID, date, and number of animals) of cattle births. The reports of TB cases were reported from lesions suggestive of mycobacterial infection detected during the official inspection of the carcasses of bovines in slaughterhouses, part of the municipal and state level official surveillance system in RS. All records were from 2016.01.01 to 2018.12.31 and were provided by the Secretary of Agriculture, Livestock and Agribusiness of State of Rio Grande do Sul (SEAPA-RS). Given that confirmation of the etiology of TLL through bacteriology or molecular biology is not routinely performed, analysis is performed on all animals presenting TLLs and not on those with a confirmed infection with a causative agent of bovine tuberculosis.

### 2.2. Characterization of the Study Population and Movement Network

We mapped the density of premises that moved at least one animal (density of active nodes), animals (animal density), outgoing-movements (outgoing-movement density), and outgoing moved animals (moved animal density) by municipality using the area of the municipalities in km^2^.

In addition, we mapped the balance of animals at the municipality level, calculated as the ratio between incoming and outgoing animals from a specific municipality (animal balance). Finally, we mapped the total of TLL by municipality. All calculations involving movements excluded those directed to slaughterhouses.

### 2.3. Network Analysis

We constructed contact networks in which premises were defined as nodes and movements between premises were considered edges. The consequent contact network A was based on the movements during the study period and was represented as directed ($Ai,j\neq A_{ji}$), such that the origin (node i) and destination (node j) give each edge a specific direction. We described the static and the time series network using data from all years of study as well as by year. Parameters measured are described in Table 1.

#### 2.3.1. Static Network Description

A static network, $G=\left(V,\;E\right)$, consists of a set of nodes V and a set of edges E, where every edge connects a pair of nodes. In the considered network, edges have a direction given by trade. Mathematically, a network can be represented as an adjacency matrix A, with elements $A_{ij}=1$ if there is an edge from node i to node j and $A_{ij}=0$ otherwise.

To characterize the static network, the parameters graph density, number of nodes, number of edges, diameter, number of moved animals, giant strongly connected component (GSCC) size and percentage, GWCC size and percentage, clustering coefficient, and mean of the shortest paths were summarized by year and for all the period of study.

##### Components Description

The real-world directed networks often exhibit a bow-tie similar structure, used as a large-scale map of the worldwide web [5,26], where the GWCC is divided into five categories with a central bulk defined as the giant strongly connected component (GSCC), where any node can reach any other node following a directed path, a component upstream to the GSCC, called in-component (GIC), where nodes can reach nodes of the GSCC following a directed path, but cannot be reached by the nodes of the GSCC; reciprocally, some nodes are downstream to the GSCC: they can be reached from the GSCC following a directed path, but cannot reach it—these constitute the out-component (GOC). The remaining nodes of the giant component are part of structures called tendrils (going out from the in-component without reaching the GSCC, or going in the out-component without coming from the GSCC) and tubes (connecting directly the in- to the out-component, without going through the GSCC) [5,26,27].

The premises were grouped according to the type of component and the distribution of the number of out-going contact chain (OCC) for each node from all study periods was calculated.

#### 2.3.2. Time Series Network Analysis

A temporal snapshots network was considered, $g\;=\;\left(V,E,T\right)$, where V is a set of nodes and E is a set of edges in each observation period T. Each edge in E is given by a triplet $\left(i,\;j,\;T\right)$ and connects node i and node j at time T. To describe the temporal patterns over time, we used a monthly time-window or series of snapshots [28]. The descriptive assessment of the temporal network snapshots was made with the same parameters described in Table 1.

### 2.4. Relationship between the Observed Network and the Distribution of TB-Like Lesion Cases

To evaluate whether there was a significant relationship between the observed network, the spatial location of the premises, and the distribution of cases (TLL) across the network, a permutation-based approach, the so-called k-test, was performed. In this test, the observed distribution of the TLL cases (*n* = 2945) in the movement network and geographic space was compared independently with the expected under the null hypothesis of random distribution of cases in space and within the network [29].

The null hypothesis was generated through 1000 Monte Carlo simulations in which the cases in the network are permutated. Reallocation of premises was performed using a degree-swapping approach to preserve the overall degree distribution of premises while randomizing the locations of TLL-premises relative to one another. TLL-premises were randomly swapped with premises whose degree fell in the same quartile of the overall degree distribution, as observed elsewhere [29,30]. The *k*-test was performed considering premises located within 5 and 10 km distances and one step (*k* = 1) within the network and was run using the number of cases reported for each year separately and for the cumulative time period.

Besides, the presence of purely spatial clusters was assessed using the spatial scan statistic with the SaTScan™ software developed by Kulldorff [31]. The number of animals with TLL in each premise was assumed to follow a Poisson distribution in which the total number of animals in each premises was the population at risk, and the observed number of cases falling into windows of increasing size was compared to the distribution of expected cases if the spatial distribution of cases occurred at random (null hypothesis). For the control, premises that sent animals to slaughterhouses and did not report TLL were used. The distribution and statistical significance of the clusters was assessed by means of Monte Carlo replication of datasets under the null hypothesis with 999 replications to ensure an adequate power for defining clusters (http://www.satscan.org accessed on 01/19/2021).

### 2.5. Disease Spread Model

We used the modelling framework SimInf, described by [32], to simulate a potential disease spread on the network explicitly over time. For this, we used a compartmental Susceptible-Occult-Reactive-Infectious (SORI) model in which the animal population was divided into four mutually exclusive compartments: susceptible $\left(S\right)$, occult $\left(O\right)$, reactive $\left(R\right)$, and infectious $\left(I\right)$.

Occult, O, state is a latent period of duration, $\lambda_{1}$, in which even though infected, animals are not detectable by antemortem TB tests and are not infectious. The animals then progress to the reactive state (R) at a rate of $\frac{1}{\lambda_{1}}$. The reactive animals are not infectious but could be detected via intradermal tuberculin testing. Reactive animals ultimately move into the infectious state (I) at a rate of $\frac{1}{\lambda_{2}}$, where $\lambda_{2}$ is the average duration of the reactive state, R. The final compartment represents animals that become infectious (I) while remaining detectable by antemortem diagnostic tests [22,33].

Once in the I stage, animals were assumed to stay infectious until culled. Animals were allocated into metapopulations (premises) and assumed to move from S to O, R, and I according to state-specific transition rates. Here, we assumed a homogenous mixing population within the premises, where all animals have identical rates of TB-causing contacts. We ignored heterogeneities related to age, space, or behavioral aspects.

The model simulated disease spread at two levels (local and global), with the local level working as a stochastic compartment model and formulated as a continuous-time Markov chain (CTMC). The Gillespie stochastic simulation algorithm (SSA) was applied to simulate the number of individuals within each compartment through time using a transition rate from $S\overset{\beta }{\to }O$ $\overset{1/\lambda_{1}}{\to }R$ $\overset{1/\lambda_{2}}{\to }I$, as

(1) $$\frac{dS}{dt}=u_{i,t}-v_{i,t}-\frac{\beta S_{i}I_{i}}{S_{i}+O_{i}+R_{i}+I_{i}}$$

(2) $$\frac{dO}{dt}=\frac{\beta S_{i}I_{i}}{S_{i}+O_{i}+R_{i}+I_{i}}-v_{i,t}O_{i}-\left(O_{i}\;\frac{1}{\lambda_{1}}\right)$$

(3) $$\frac{dR}{dt}=\left(\;O_{i}\frac{1}{\lambda_{1}}\right)-\left(R_{i}\frac{1}{\lambda_{2}}\;\right)-v_{i,t}R_{i}$$

(4) $$\frac{dI}{dt}=\;R_{i}\frac{1}{\lambda_{2}}\;v_{i,t}R_{i}$$

in a specific time t [34]. The global level was incorporated assuming a directed temporal network $g\;=\;\left(V,E,t\right)$ using the day-to-day scheduled movements from 1 January 2016 to 31 December 2018, where the number of incoming and outgoing animals update the number of animals in a given node i at time t. The movements with destination to slaughterhouses were considered by subtracting these animals from the simulations, and the number of eligible animals to move within nodes were sampled randomly. To maintain a stable herd size, the number of new animals due to births, $u_{i,t}$, were incorporated using the day-to-day scheduled births based on the farm owners’ declaration to the official surveillance system for each specific premises, and v is the day-to-day scheduled movements to slaughterhouses in a specific premises i at specific time t.

The number of animals present on each premises in December 2016 was considered the herd size. We found some premises with negative balance in the herd population after considering in/out animal movements within premises, births, and deaths records. To correct this balance, the negative number of animals were restored before we ran the model.

#### 2.5.1. Parameters of the Model

Since we do not have field data for RS or Brazil to calculate the parameters, we used parameters reported in models and studies of TB at the farm level. For the within-premises transmission coefficient rate, β (per cow and year), we alternatively used the following values: 2.23 [35], 2.76 [12,22,33,36,37,38,39], and 5.2 [12,22,37,38]. For the other transmission rates, we assumed $\lambda_{1}$ = 41 days [33,40,41] and $\lambda_{2}$ = 6 months [12,33,36,38,41].

To run the model, we considered two initial scenarios: the first one using 3055 premises randomly sampled from the network in each simulation as infected. The second scenario was started considering as infected the 3055 premises in which at least one animal had TB-like lesions reported at slaughterhouses during the 2016–2018 period. For both models, the simulation assumed an initial within-premises animal prevalence of 7% according with the intra-herd TB prevalence reported in Reference [16].

#### 2.5.2. Disease Spread and Targeted Control Action Modeling

The models were run using 1000 replications for each different targeted intervention measure by sequentially removing an increasing number from 1 to 25,000 nodes prior to the simulation based on network parameters rank: degree, betweenness, PageRank, and random nodes. All network-based parameters were calculated using the full contact network, and after each node-removal, the epidemic curve and prevalence were calculated and the reduction of the number of infected farms was calculated at the end of each simulated scenario (simulation day = 1095). Additionally, we also simulated a scenario in which no control measures at all were applied (No-control). To explore significant differences on the final number of infected farms between scenarios, the Kruskal–Wallis and Wilcoxon tests followed by post-hoc tests using Bonferroni corrections were used.

#### 2.5.3. Sensitivity Analyses

Since we proposed a model with local and global dynamics, the sensitivity analyses were performed separately at the local level (only one node was simulated) and the global level (considering the whole population). At the local level, the average incoming and outgoing animals were calculated from the movement data as a six-month rate in order to account for the herd-level dynamics; therefore, 7% of animals in the simulated herd were added and removed every six months. The sensitivity analysis consisted of 100 replications of 1000 Latin Hypercube Sampling (LHS), and the impact of varying parameters (see below) was measured by comparing the final prevalence of infection in the herd. For the global sensitivity analysis, the whole network was considered, and the outcome of varying the same parameters was assessed by comparing the number of infected premises at the end of simulations using 300 LHS and 100 replications.

The following values were considered for the parameters governing disease transmission based on available literature: β (1 to 5.2) [12,22,33,35], $\lambda_{1}$ (1/14.1 to 1/45) [12,22,33,39,42], and $\lambda_{2}$ (1/180 to 1/630) [12,22,33,38,39,42]. For the local sensitivity analysis, different herd sizes (10 to 1000 animals) were also considered. The LHS were used to explore the parameters space [43] and the partial rank correlation coefficients (PRCCs) were considered to measure the strength of the correlation between the modified parameter and the outcome: a positive PRCCs value indicates that the value of the model output can be increased by increasing the respective model input parameter, and the model output can be decreased by forcing down the relative input parameter. Otherwise, a negative PRCCs value indicates a negative correlation between the model input and output [43,44,45]. In general, the PRCCs-LHS provides a measure of monotonicity between a set of parameters and the number of infected animals/infected premises after removal of the linear effects of all parameters except the parameter of interest [46,47].

#### 2.5.4. Software

The software used for analysis and graphics was R statistical software (v. 3.6) [48] with RStudio editor using the packages: igraph 1.2.4 [49], tidyverse 1.2.1 [50], SimInf 6.3.0 [32], sf 0.5–3 [51], brazilmaps 0.1.0 [52], incidence [53], and epiR [54], and for the spatial cluster analysis, SaTScan™ software was used [55].

## 3. Results

### 3.1. Characterization of the Study Population and Movement Network

**Figure 1:**
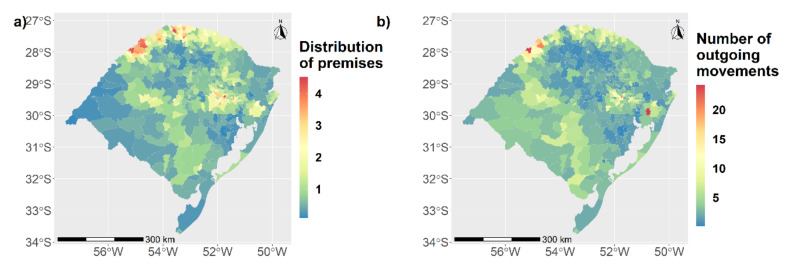
Spatial distribution of premises and animal movements in Rio Grande do Sul (RS) state. (**a**) Distribution of premises in km^2^ by municipalities. (**b**) Number of outgoing movements in km^2^ by municipalities.

The municipalities with the largest number of premises (3 to 4.51 premises/km^2^) were located in the northern and in the central-western parts of the state near the metropolitan region of Porto Alegre (Figure 1a).

The higher number of outgoing movements was localized in the southwestern part of the state, with zero to 20 cattle movements/km^2^ (green to yellow areas in Figure 1b), and there was also a hotspot in the central-western part with 24.19/km^2^ outgoing movements.

**Figure 2:**
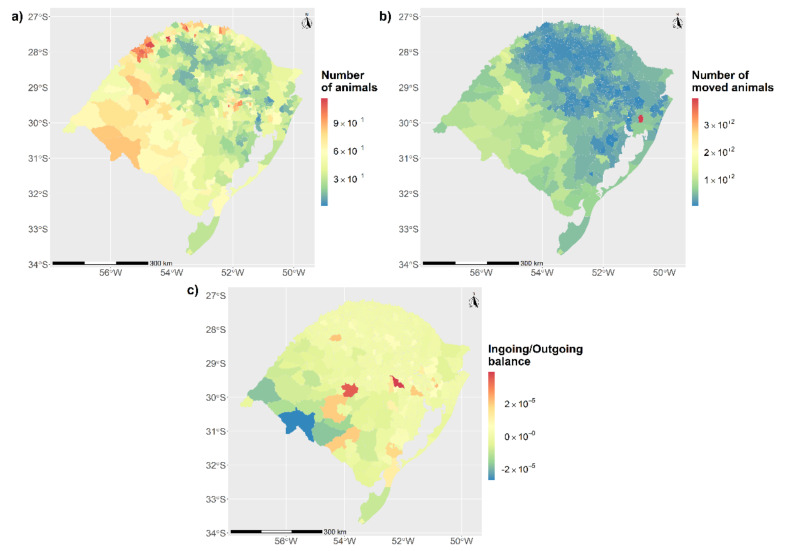
Spatial distribution of animals, outgoing movements of animals, and ingoing/outgoing movements balance in RS state. (**a**) Herd size by municipalities by km^2^. (**b**) Number of outgoing number of animals in km^2^ by municipalities. (**c**) Balance between ingoing/outgoing total number of animals.

The number of animals by km^2^ showed a heterogeneous distribution, with a higher cattle population in the southwestern part of the state (60 to 115 animals/km^2^) (Figure 2a). This region also had the highest number of moved animals (46 to 392 moved animals/km^2^) (Figure 2b). In general, the cattle trade had a neutral ingoing/outgoing balance in the total number of the animals traded, with the exception of the south-west region, which exported more than they imported, and two municipalities with more than 300,000 imported animals (Figure 2c).

All animal movements were grouped into six purpose categories: movements of animals to fattening units (Fattening), including 56.7% of all movements, animals destined for a Semen Collection and Processing Center, animals destined for natural breeding on a specific property or other reproductive activities (Reproduction), with 5.62%, movements to slaughterhouses (Slaughterhouses) with 36.7%, movements to or from events or fairs (to events (0.03%) and return from events (0.06%), respectively), and movements for other purposes (veterinary care, weighting, etc., others (0.086%)). The monthly number of movements by purposes are depicted in the Supplementary Figure S1.

#### 3.1.1. Static Network Description

The annual number of nodes, edges, and animals and the estimates for network parameters graph density, GSCC, and GWCC throughout the 3-year span are shown in Table 2. The number of animals moved increased over the years but no trend in the number of edges was observed over the study period. Despite the observed decrease in the number of nodes, an increase in graph density was detected. The increase in the diameter of the network together with the mean of shortest paths showed a more connected network year by year (Table 2).

#### 3.1.2. Components Analysis

**Figure 3:**
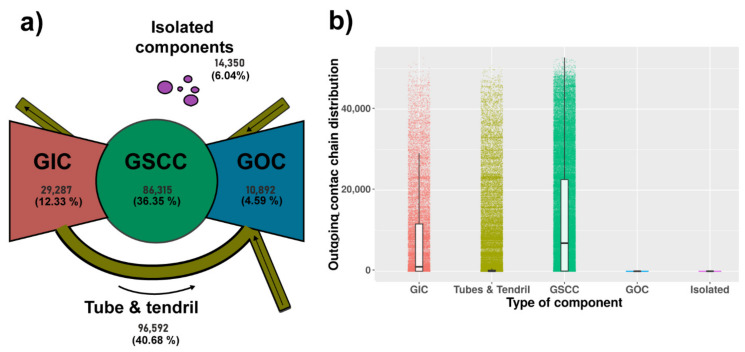
(**a**) Bow-tie structure of a directed graph. The GWCC was separated into GSCC, the relative in- and out-components, tendrils, and tubes. Adapted from References [5,26]. (**b**) Each little point represents the outgoing contact chain distribution by type of component for all study periods, the white boxes represent a boxplot of components distributions.

To represent the results, the static network was depicted using a so-called bow-tie partition (Figure 3a), where nodes are classified into four different components: giant in component (GIC), giant out component (GOC), tubes and tendrils, and isolated components. The Figure 3a shows the number and proportion of the different components, with the tube and tendril component showing the highest size (40.7%) followed by the GSCC component (36.35%). The size of the GWCC was 223,086 and represented 93% of the network.

We calculated the reachability of the directed network using the thoughtful static network components description. The spreading potential was calculated using the size of the outgoing contact chain in each component in the network, the GIC and GSCC showed a higher outgoing contact chain distribution compared with GOC and isolated, and the tubes and tendril had a lower distribution despite a higher number of outliers (Figure 3b).

### 3.2. Time Series Network Description

**Figure 4:**
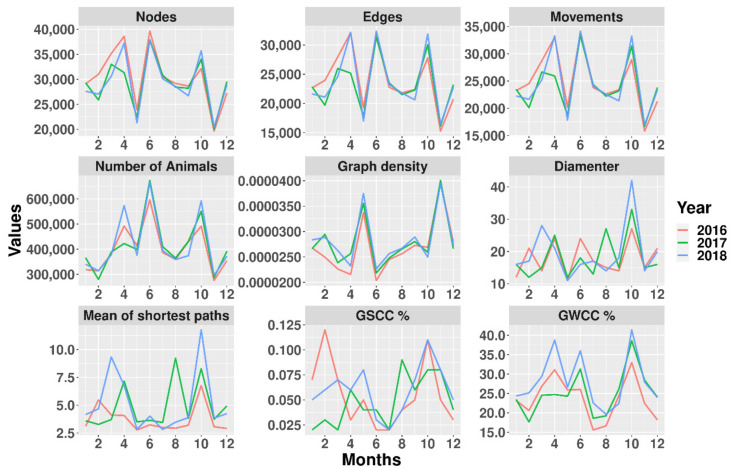
Description of the network using a time-aggregate windows of 30 days from the parameters: nodes, edges, movements, number of animals, graph density, diameter, mean of the shortest paths, GSCC, and GWCC. The description of parameters are shown in Table 1.

When the network parameters were analyzed on a monthly basis, a seasonal trend was observed, with marked maximum and minimum values, especially for the months 5 and 11 (May and November). The number of animals, graph density, and GSCC/GWCC were strongly influenced by the decreasing number of nodes and edges (Figure 4).

#### 3.2.1. Relation between the Observed Network and the Distribution of Cases across the Network

**Figure 5:**
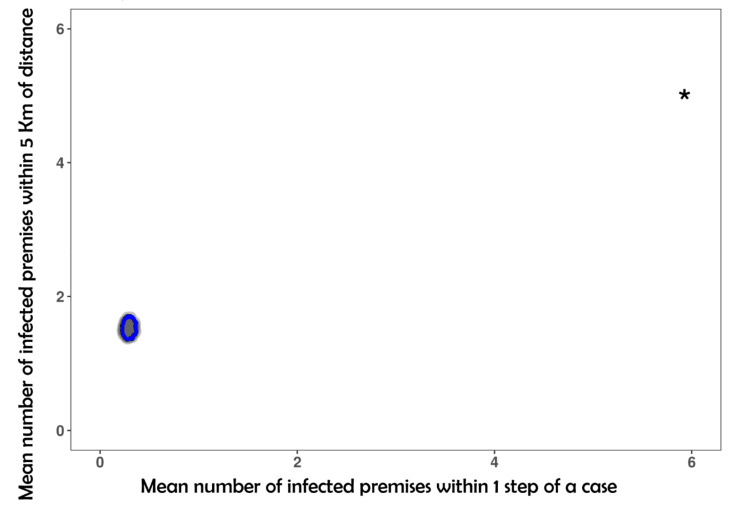
Graphical results of the k-test for the cumulative network considering a threshold of 5 km and k = 1 step in the network. The star indicates the observed mean of positive premises within 1 step in the network (x-axis) and positive premises within 5 km (y-axis). The grey-shaded region represents the null distribution of the k-statistic when positive premises were randomly distributed within the network. Blue line represents 95% of the null distribution of positive premises within one step in the network.

The k-test showed significant clustering of TB-like lesion locations both in the annual (Figures S2 and S3) and cumulative networks (Figure 5). Premises with TB-like lesions were connected to an annual mean of 2.07, 2.44, and 2.68 positive premises in 2016, 2017, and 2018 respectively, and 5.92 for the cumulative years. These values were significantly (*p*-value < 0.001) higher than what would be expected under the null hypothesis of random distribution of lesions across the network. The observed number of premises including animals with TB-like lesions located within 5 and 10 km of other premises (annual ranges of 3.80–4.01 and 9.48–10.07, respectively) was also significantly greater (*p* < 0.001) than what would be expected if premises were distributed randomly in space (Figure 5).

#### 3.2.2. Spatial Distribution and Cluster Analysis

**Figure 6:**
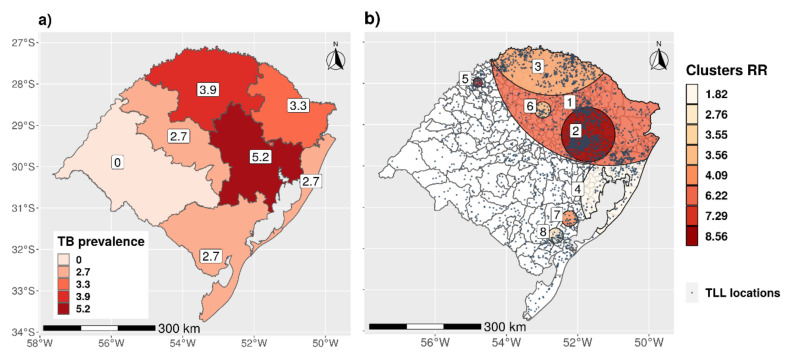
(**a**) Map of prevalence of TB by productive regions in RS (Adapted from [16]) (**b**) Clusters of TLL locations (premises with reported TLL) for the RS state for all of the study period. The blue dots represents the premises locations.

We reproduced the map by [16] (Figure 6a) with the prevalence by productive zones and compared with the clusters that were found after the analysis of the spatial distribution of the premises from which 2945 animals with TLL (out of 3055 cases located on farms with available geolocation) originated (Figure 6b).

The results of the cluster spatial analysis are shown in Table 3 and Figure 6b. Seven significant spatial clusters were found in the state. These clusters were located in the northern and eastern parts of the state with radius ranging between 12.5 to 323.17 km and relative risks between 1.82 and 8.56.

### 3.3. Disease Spread Modeling

**Figure 7:**
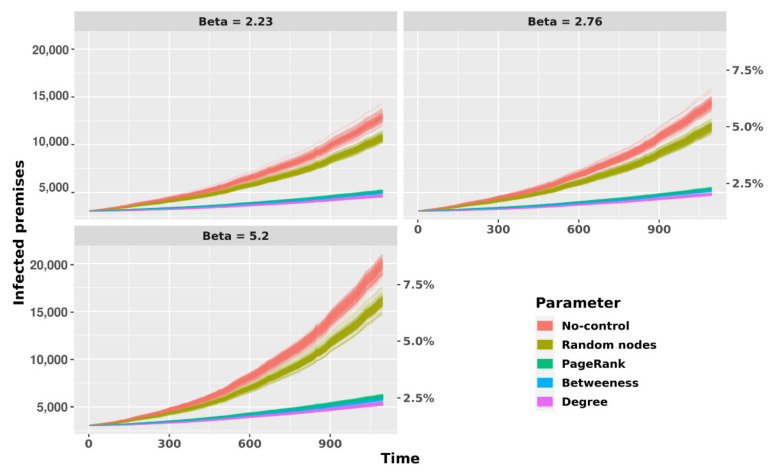
Predicted infection curves controlling by network-based control actions (removal of 25,000 premises based on different criteria) and without control action (No-control) in the temporal network representation of cattle movements for different probabilities of transmission (1, 2.76, and 5.2) using 3050 random nodes as initial infected premises in each simulation. The Y-left axis represents the total of infected premises at the end of simulation, and the y-right represent the percentage of infected premises. The x-axis represents the simulation time in days.

Results of two different sets of scenarios after 1000 model replications are described: the first one corresponds to the scenario in which no control measures are included (No-control). This was the scenario leading to the largest increase in the number of infected premises at the end of the simulated period, from 3055 herds to between 10,323 and 19,480 when the infection was seeded in random premises (Figure 7), and between 10,664 and 19,836 when infection was seeded in TLL-positive premises (Figure S5).

**Figure 8:**
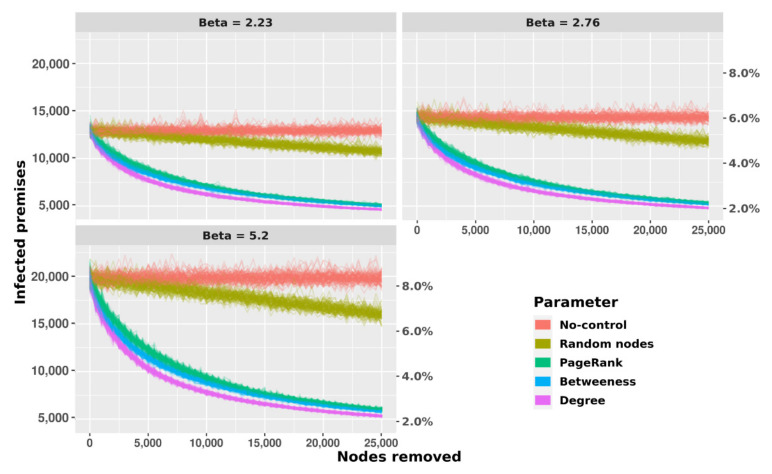
Node removal model in RS state based on the transmission rates (1, 2.76, and 5.2) and network parameters: PageRank, betweenness, degree, and random nodes from 100 model interaction using 3050 random nodes as initial infected premises in each simulation. The Y-left axis represents the total of infected premises at the end of simulation, and the Y-right represents the percentage of infected premises. The x-axis represents the simulation time in days.

In the second set of scenarios, when control actions based on network metrics (removal of up to 25,000 nodes based on the different network metrics) were simulated, a larger impact (i.e., decrease in the number of infected premises over time compared to the No-control scenario) was observed when nodes were removed based on higher degree followed by PageRank and betweenness, while removal of randomly selected nodes showed the lowest impact, as expected, with a result very close to the baseline (No-control) scenario. These patterns were similar regardless of whether the infection was seeded in randomly chosen case premises (Figure 8) or TLL-positive premises (Figure S4).

When the prevalence in the last day of simulation (day = 1095) obtained in the different scenarios was assessed, similar results were observed, and again, removing nodes based on degree had the largest impact on the infected number of premises across all transmission coefficients considered. A higher performance of the control strategy when removal was based on degree compared with the other SNA parameters was observed (*p* < 0.001, Kruskal–Wallis test and Dunn post-hoc test with Bonferroni corrections). Similarly, removal of nodes based on PageRank and betweenness showed also higher performance in reducing the prevalence by more than 50% (Figure 8 for randomly seeded case herds, Figure S5 for infection seeded in TLL-positive premises).

### 3.4. Sensitivity Analyses

**Figure 9:**
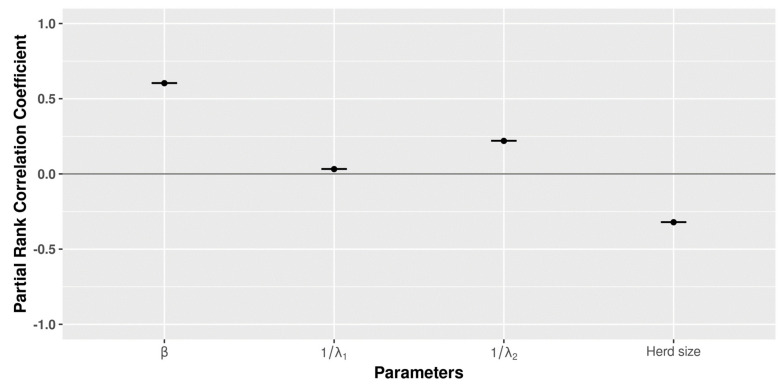
Latin Hypercube Sampling-Partial Rank Correlation Coefficients (LHS-PRCC) sensitivity analysis (1000 samples and 100 replicates) considering the impact of varying values for parameters β, $1/\lambda_{1}$, $1/\lambda_{2}$, and herd size on the within-herd prevalence with a PRCC, significantly (*p* < 0.05).

For the analysis at the local level, 1000 LHS were replicated 100 times considering the impact of variations in certain parameters (β, $1/\lambda_{1}$, $1/\lambda_{2}$, and herd size) on the prevalence of infected animals. The parameter with the highest positive influence was β, followed by $1/\lambda_{2}$, indicating that a higher value resulted in an increase of the within-herd prevalence. Herd size showed a negative correlation. All intervals in the PRCCs were very small. The results are represented graphically in Figure 9 and Figure S6.

### 3.5. Global Sensitivity

**Figure 10:**
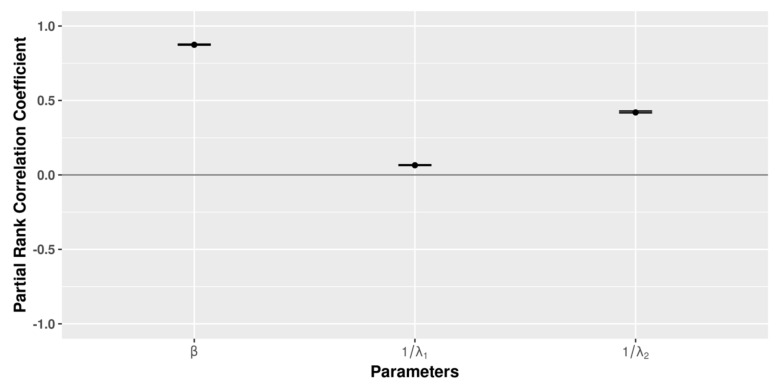
LHS− PRCC global sensitivity results for 1000 samples and 100 replicates for parameters β, $\frac{1}{\lambda_{1}}$, and $1/\lambda_{2}$ with a PRCC, significantly (*p* < 0.05).

When the sensitivity analysis was carried out at the global level, the β parameter again had the largest influence on the model outcome (number of infected farms at the end of the simulation), with other parameters showing similar correlation with the output (Figure 10). Complete results are presented in the Figure S7.

For the global level, the assessment of the number of infected premises when just one parameter (β, $\lambda_{1}$, or $\lambda_{2}$) was perturbed while the others remained fixed demonstrates a strong impact of all three in the predictions, with overall simulated infected premises values ranging between 3.71% and 8.01% depending on the parameter. The β parameter showed the highest widespread intervals of the final range prevalence; in addition, a wider range as also observed for the parameter $\lambda_{2}$, this can be expected due to the higher range of this parameter (from 3 to 21 months). All results are depicted in the Figure S8.

## 4. Discussion

This work explored the cattle trade in RS state and its possible association with the distribution of TB in herds using the presence of animals with TLL as an indicator. This was done through a description of the static network, density of animals, and movement patterns. Besides, monthly snapshots were generated to represent the temporal trends in the network over time to give an overview of the cattle trade in the RS state. We then explored the existence of a plausible association between the positive TLL premises and their network connections and geographical proximity. Finally, TB spread was simulated using a SORI model on the characterized network to explore the impact of network-based targeted surveillance and control in RS state in Brazil.

The use of the static and time series snapshot network was aimed at providing an overview of the topology and structure of the network. In the RS state, the surveillance system is based on intervention at the municipality level. For this reason, we described the spatial patterns at the municipality level. However, to avoid the possible distortion due to the heterogenous size of the municipalities, we adjusted the results considering a density in km^2^. This analysis showed a higher concentration of premises in the northwest part of the state but a higher number of movements and animals in the south part. These patterns are probably due to the higher herd sizes of premises in the southwest and southeast part of the state compared with the other regions. This is particularly important because these regions share a large frontier with Uruguay and Argentina, and these south regions were also identified as risk areas for foot and mouth disease [56].

When we explored the monthly temporal network, we found a clear temporal trend between May and November, when the trade activity decreased considerably. These changes over time can be explained by the vaccination campaigns against foot and mouth disease, which are applied during these months when many batches are confined for the count and vaccination [7].

We explored the connected components using a bow-tie description as adopted in Reference [5]. This structure can be used as a proxy of the potential risk of spread due to certain premises according to their location in the bow-tie structure. As expected, the GSCC showed a higher spread potential considering the OCC distribution (Figure 5) despite the lower number of premises in the GIC (12.33% of the network). Tubes and tendrils also showed higher outlier values of OCC, but it is worth noting that this component also has a greater number of premises on the network (40.68% of the network). The premises at the end of the commercial trade were allocated in the GOC, showing lower OCC values.

We explored the distribution of the TLL-positive premises over the components and we found 61.9% of the cases in the GSCC, which could explain in part the higher output values at the end of simulations in the SORI model simulation when these TLLs were used as index cases (Figures S5 and S6), followed by tube and tendril, GOC, GIC, and isolated, with 16.6%, 5.67%, 5.19%, and 1.01%, respectively. Therefore, a large percentage of cases have a high potential to be disease spreaders.

Considering these results, we calculated whether the pattern of TLL cases within the observed contact network was likely to have resulted from transmission through animal movements or through contact between neighboring premises. The k-test showed a significant association between TLL-positive premises and the structure of the network in the network of each year (2016 to 2018) and the full period, so that premises with animals with TLL tended to be closer in the network over the expected values. This association was also present in space using threshold distances of 5 and 10 km, so that premises with TLL animals were closer in space than expected. Similar results were obtained in Uruguay [29], which shares a long border with RS.

The results of the local spatial clustering analysis confirmed these results and led to the identification of eight clusters (with relative risks ranging between 1.8 and 8.6). These high-risk zones are largely consistent with areas with a high TB prevalence in RS reported in 2016 [16] (see Figure 6). Several factors could explain this apparent higher risk in the spatial clusters, including different herd sizes (herds in risk areas were significantly (Mann–Whitney test, *p* < 0.01) smaller (median = 16 animals) than those outside of risk areas (median = 80 animals)), which could be associated with different management of premises, and higher density of animals and premises (Figure 2a,b), especially for dairy cattle.

Since we showed the association between TLL-positive premises in the network, a SORI model was used to evaluate the possible spread of the infection through cattle movement under the premise of no testing and no movements restrictions, and the impact of several control actions based on removal of certain nodes according to their relevance in the network. Once we simulated the removal of 15,000 nodes (6.6% of all nodes in the network) based on degree, betweenness, and PageRank, we obtained a reduction in the prevalence at the end of the study period of around 50% for all transmission rates. These results agree with other node remotion models based on network approaches using network fragmentation of the connected components in Italy, Great Britain, and Cameroon, suggesting that the SNA parameter degree can be used for selection of nodes to be subjected to control measures in scale-free networks [57,58,59]. The number of infected premises at the end of the simulation when seeding the infection in farms with TLL was higher than when using random nodes. This difference can be explained in part by the location of the TLL-positive premises in the network structure, since 56% of them were in the GSCC of the network, and thus had more reachability.

We also explored if the order of the movements had an impact on the simulated disease spread (methodology detailed in the Supplementary Material), and for that reason, we used a permutated network in which the order of the movements was changed randomly. Results showed a significant increase in the number of infected premises in the permuted networks (*p*-value < 0.001), with a median of the prevalence in 6.4% versus 5.9% for real-data simulations, suggesting that the infection curves are highly dependent on the order of the connections despite the degree distribution (Figure S9).

In this study, data on the confirmation of laboratory results were not available, and therefore we could not be completely sure that farms with TLL animals were truly infected. Moreover, given the lack of ante-mortem test results in all farms and limitations in the sensitivity of abattoir surveillance [60], other farms could have been infected but remain undetected.

For these reasons, we selected random premises as cases as our starting point in part of our modeling exercises. Interestingly, the patterns in the results were relatively similar to those obtained when seeding the infection in farms with TLL animals, although a much larger variability in the results was observed, as expected, suggesting that the initial farms being infected could in fact impact the outcome of the transmission over a three-year period.

Nevertheless, in a study conducted in 2015 in RS, the Comparative Cervical Test revealed 26.4% of reactors and 13.2% of inconclusive animals in 53 dairy cattle premises. At slaughter, TB-like lesions were found in tissues of 92.9% and 71.4% of the reactor and inconclusive animals [61], indicating that the use of TLL can be a good proxy of the infected animals in this region. However, an important limitation of the official veterinary system is the absence of routine confirmation of these TLL reported in slaughterhouses. One study reported the confirmation of *Mycobacterium bovis* infection in 28 out of 1233 TLL, but no information on how many of the 1233 samples were actually subjected to bacteriological analyses was reported [62]. Another work reported 253 laboratory confirmations out of 587 TLL collected in slaughterhouses in another state of Brazil from 2002 to 2004 [63]. Therefore, the results obtained from the description of these findings in slaughterhouses should be interpreted with caution.

Therefore, we are confident that this work provides useful information for the control and eradication program of tuberculosis of the Brazilian government (PNCEBT). For this work, we were not able to determine the number and the impact of illegal or unreported movements, so it was assumed that the spread of infection was only due to known movements, neglecting other potential pathways such as movements of other species susceptible to TB, movement of owners, workers, or veterinarians, direct contact between neighboring farms (fence-to-fence contact), and transmission via fomites [27]. Thus, the control of TB and other infectious diseases must be analyzed in conjunction with other epidemiological tools and approaches.

## 5. Conclusions

The characterization of the network provided detailed information to understand the cattle trade, spatial patterns, and describe the temporal trading fluctuations, suggesting that surveillance activities should consider the spatiotemporal variation. This network characterization may result valuable to quantify the network-associated risk for other transmissible diseases through cattle movements. There was a significant relationship between the observed network, the spatial location of the premises, and the distribution of cases (TLL), so tracking origin and destination of animals from TLL-positive premises can be useful to detect other infected animals/premises in the network. The SORI model developed can be used to estimate the epidemic sizes of TB in RS state considering the contact trade, may help to determine the network metrics that should be selected for risk-based selection of target premises according to the approximation of epidemic sizes, and it is useful to evaluate the impact of node removal to calculate the minimum number of premises to be targeted.

## Figures and Tables

**Table 1:** Description of network analysis terminology and metrics.

Parameter	Definition	Reference
Nodes	The unit of interest in network analysis, for example, premises or slaughterhouses.	[23]
Edge	Link between two nodes in the network.	
Degree (k)	Number of unique contacts to and from a specific premise. When the directionality is considered, the ingoing and outgoing contacts are defined: out-degree is the number of contacts originating from a specific premise, and in-degree is the number of contacts coming into a specific premise.	
Movements	The number of animal movements records over a certain period of time.	
PageRank	Google PageRank measure, a link analysis algorithm that produces a ranking based on the importance for all nodes in a network with a range of values between zero and one. The PageRank calculation considers the indegree of a given premise and the indegree of its neighbors.	[24]
Betweenness	The extent to which a node lies on paths connecting other pairs of nodes, defined by the number of geodesics (shortest paths) going through a node.	
Clustering coefficient	Measures the degree to which nodes in a network tend to cluster together (i.e., if A → B and B → C, what is the probability that A → C), with a range of values between zero and one.	
Giant weakly connected component (GWCC)	Proportion of nodes that are connected in the largest component when directionality of movement is ignored.	[23]
Giant strongly connected component (GSCC).	Proportion of the nodes that are connected in the largest component when directionality of movement is considered.	[23]
Out-Going Contact chain (OCC)	The outgoing contact chain (OCC) quantifies the number of ‘downstream’ premises that could potentially acquire infection from the index premises through outgoing animal movements, adhering to the chronological order of the movements.	[5,25]

**Table 2:** Static network description. Network parameters of RS state by year and for all years.

Year	Nodes	Edges	Animals	Graph Density	Diameter	GSCC (%)	GWCC (%)	Clustering Coefficient	Mean Shortest Paths
2016	158,469	316,455	4,809,197	1.26 × 10^−5^	40	29,148 (18.39)	133,069 (83.97)	0.021	10.71
2017	151,498	307,943	4,960,304	1.34 × 10^−5^	45	27,326 (18.04)	125,685 (82.96)	0.020	10.29
2018	149,754	314,282	5,030,184	1.40 × 10^−5^	35	26,797 (17.89)	126,709 (84.61)	0.017	9.86
All years	237,436	938,680	14,799,685	1.67 × 10^−5^	32	86,315 (36.35)	223,086 (93.96)	0.025	8.13

**Table 3:** Significant high rate TLL spatial clusters in Rio Grande do Sul area.

			No. of Cases in Cluster			
Cluster	Radius (km)	No. of Herds in Cluster	Observed	Expected	Relative Risk	*p*-Value
4	123.55	3629	257	146.91	1.82	3.18 × 10^−11^
8	20.47	418	52	19.1	2.76	0.000152
6	21.95	1406	43	12.23	3.55	3.44 × 10^−6^
3	197.9	21,766	517	167.34	3.56	1.00 × 10^−17^
7	21.04	1180	30	7.39	4.09	0.000134
1	328.17	55,109	1854	662.12	6.22	1.00 × 10^−17^
5	12.5	1134	32	4.43	7.29	3.52 × 10^−11^
2	73	15,744	801	124.82	8.56	1.00 × 10^−17^

## Data Availability

The data that support the findings of this study are available from the official veterinary office upon reasonable request. Restrictions apply to the availability of these data, which were used under confidentiality agreements.

## Supplementary Materials

The following are available online at https://www.mdpi.com/2076-2607/9/2/227/s1, Figure S1: Monthly number of outgoing movements by the declared purpose from all study periods in RS state, Brazil. Figure S2: Graphical results of the k-test. The grey-shaded region in the graph represents the null distribution of the k-statistic when positive premises were randomly distributed within the network. Bold line represents 95% of the distribution of positive premises within 1 step. The star indicates the observed mean of positive premises within 1 step in the network (x-axis) and positive premises within 5 km (y-axis). Figure S3: Graphical results of the k-test. The grey-shaded region in the graph represents the null distribution of the k-statistic when positive premises were randomly distributed within the network. Bold line represents the 95% of the distribution of positive premises within 1 step. Figure S4: Infection curves controlling by network-based control actions and without control action (No-control) in the temporal network representation of cattle movements for different probabilities of transmission (1, 2.76, and 4.13) using TLL-positive premises from 2016 to 2017 as initial infected premises. Figure S5: Node removal model in RS state based on the transmission rates (1, 2.76, and 4.13) and network parameters: PageRank, betweennesses, degree, and random nodes from 10 model interactions using 3050 TLL-positive nodes as initial infected premises in each simulation. Figure S6: (a) Local sensitivity analysis, dot colors represent the mean of prevalence in the stochastic outputs model by the parameters: Herd size, beta (β), and 1/lambda 2 (1/λ). (b) Local sensitivity analysis, dot colors represent the mean of prevalence in the stochastic outputs model by the parameters: 1/lambda 1 (1/λ), beta (β), and 1/lambda 2 (1/λ). Figure S7: Global Sensitivity analysis by parameters. Each dot represents one simulation of 1000 for the different parameter values (β, λ_1, and λ_2). Figure S8: Global permutation sensitivity analysis, between the permuted networks and the real-data network. Figure S9: Model sensitivity of global dynamics analysis, dot colors represent the mean of infected premises in the stochastic outputs model by the parameters: 1/lambda 1 (1/λ), beta (β), and 1/lambda 2 (1/λ).
