# Supplementary figures and images for: Use of Network Analysis and Spread Models to Target Control Actions for Bovine Tuberculosis in a State from Brazil
Source: Microorganisms. 2021 Jan 22;9(2):227. doi: 10.3390/microorganisms9020227 (PMC7912437; doi:10.3390/microorganisms9020227)

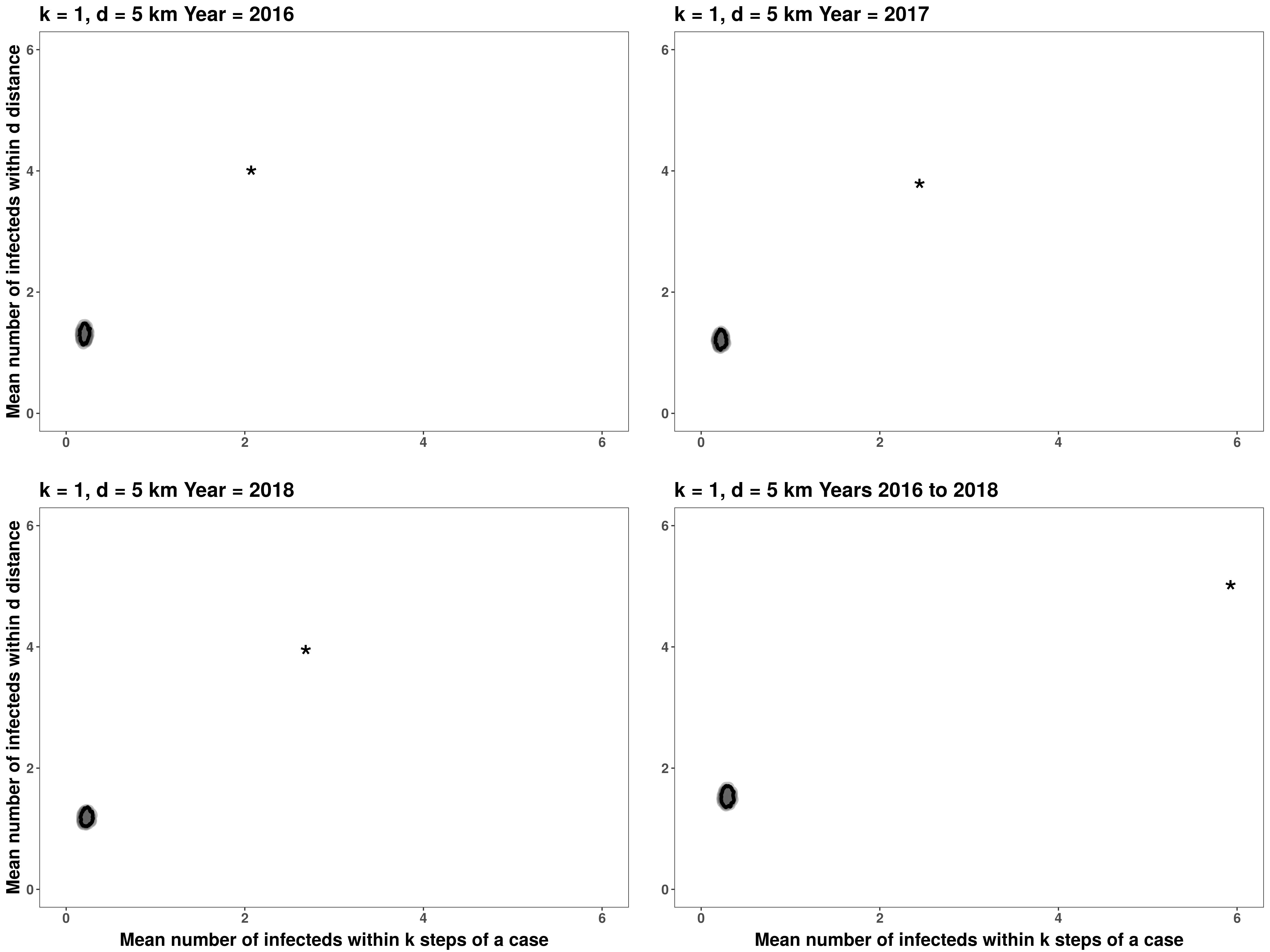

Supplement: Supplementary file 1 [file microorganisms-09-00227-s001.zip › figuras suplementar/Supplementary figure 2 ktesp_5km_comp.png]

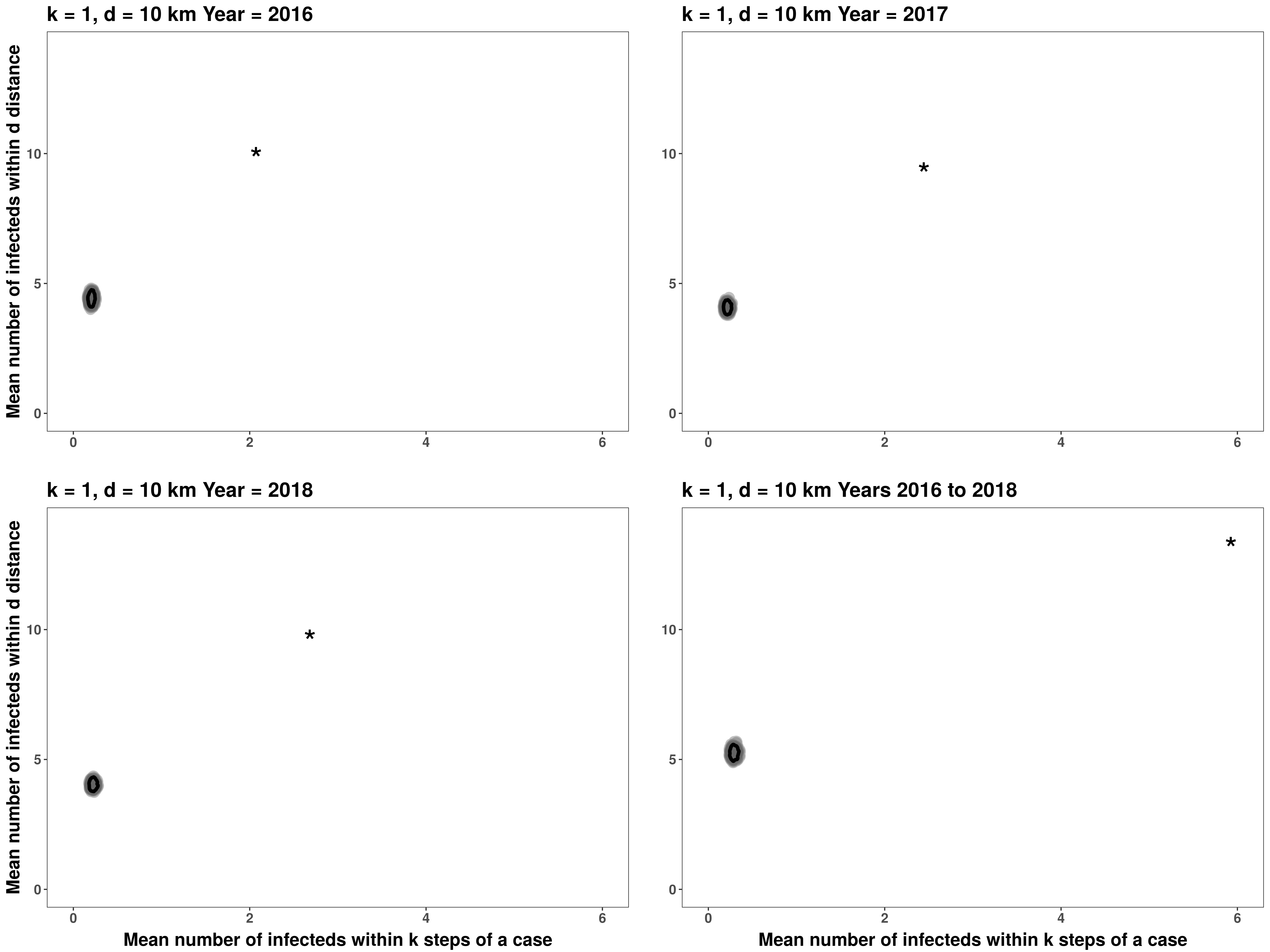

Supplement: Supplementary file 1 [file microorganisms-09-00227-s001.zip › figuras suplementar/Supplementary figure 3 ktesp_10km_comp.png]

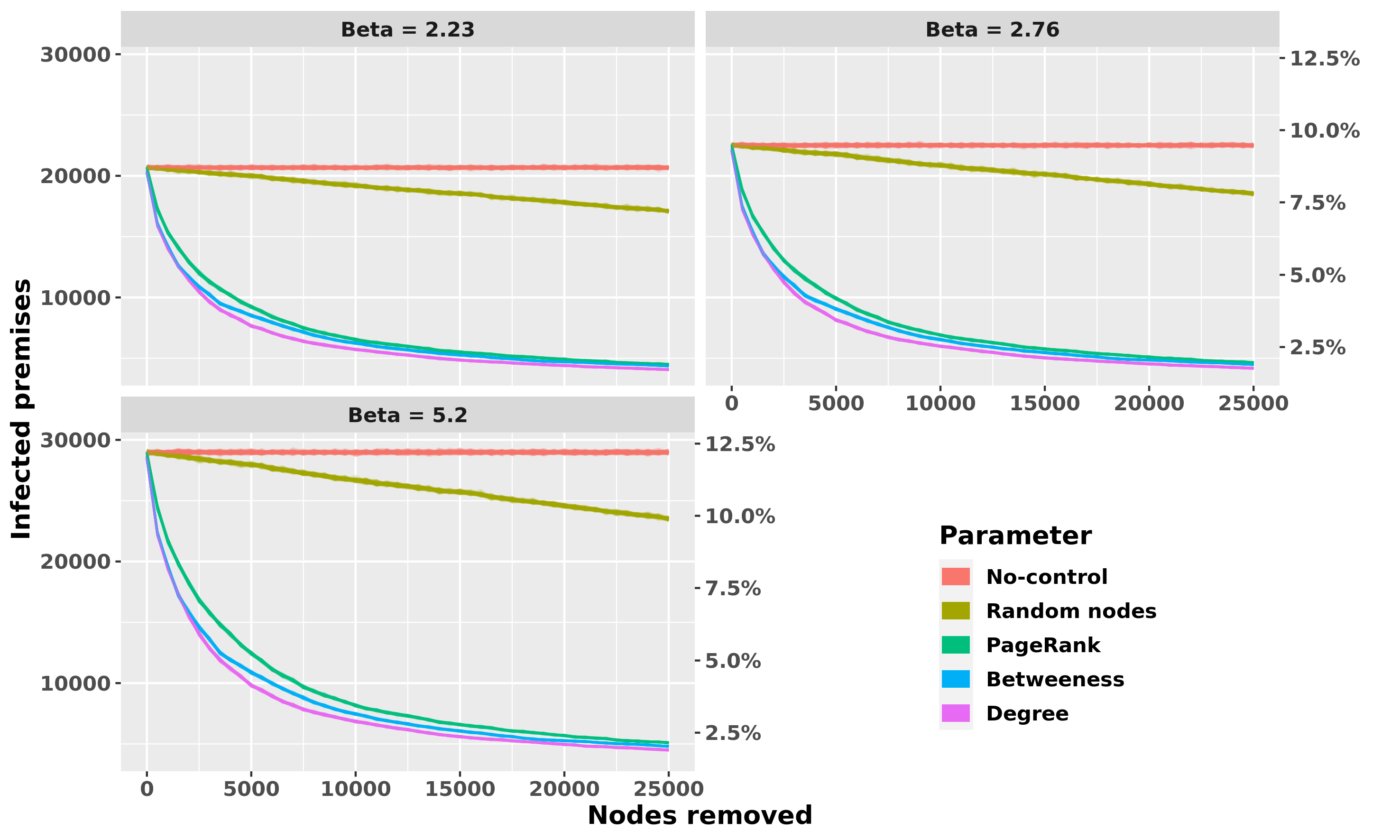

Supplement: Supplementary file 1 [file microorganisms-09-00227-s001.zip › figuras suplementar/Supplementary figure 5 TLS_sori_removal_simulation_tll2.png]

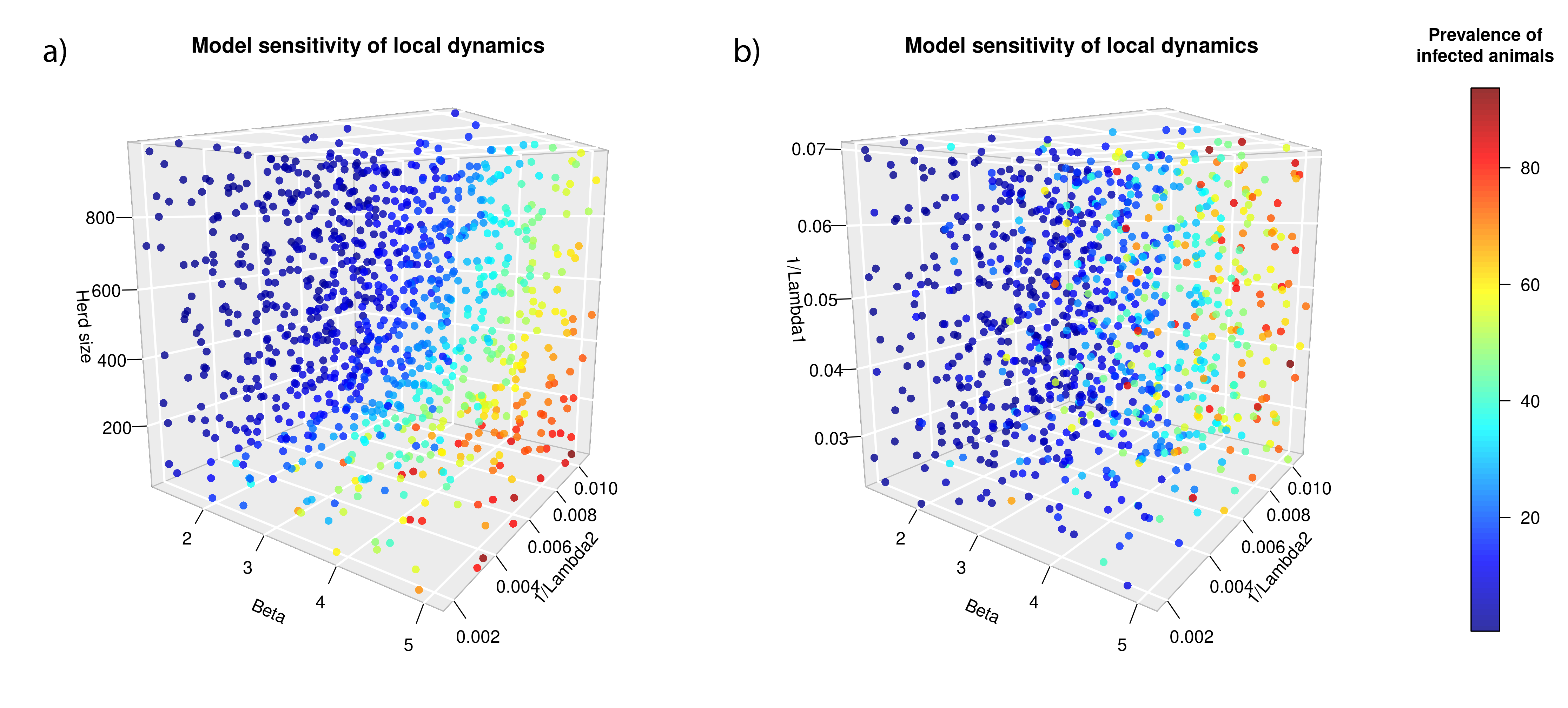

Supplement: Supplementary file 1 [file microorganisms-09-00227-s001.zip › figuras suplementar/Supplementary figure 6 sensiblidade LOCAL model sori-02.png]

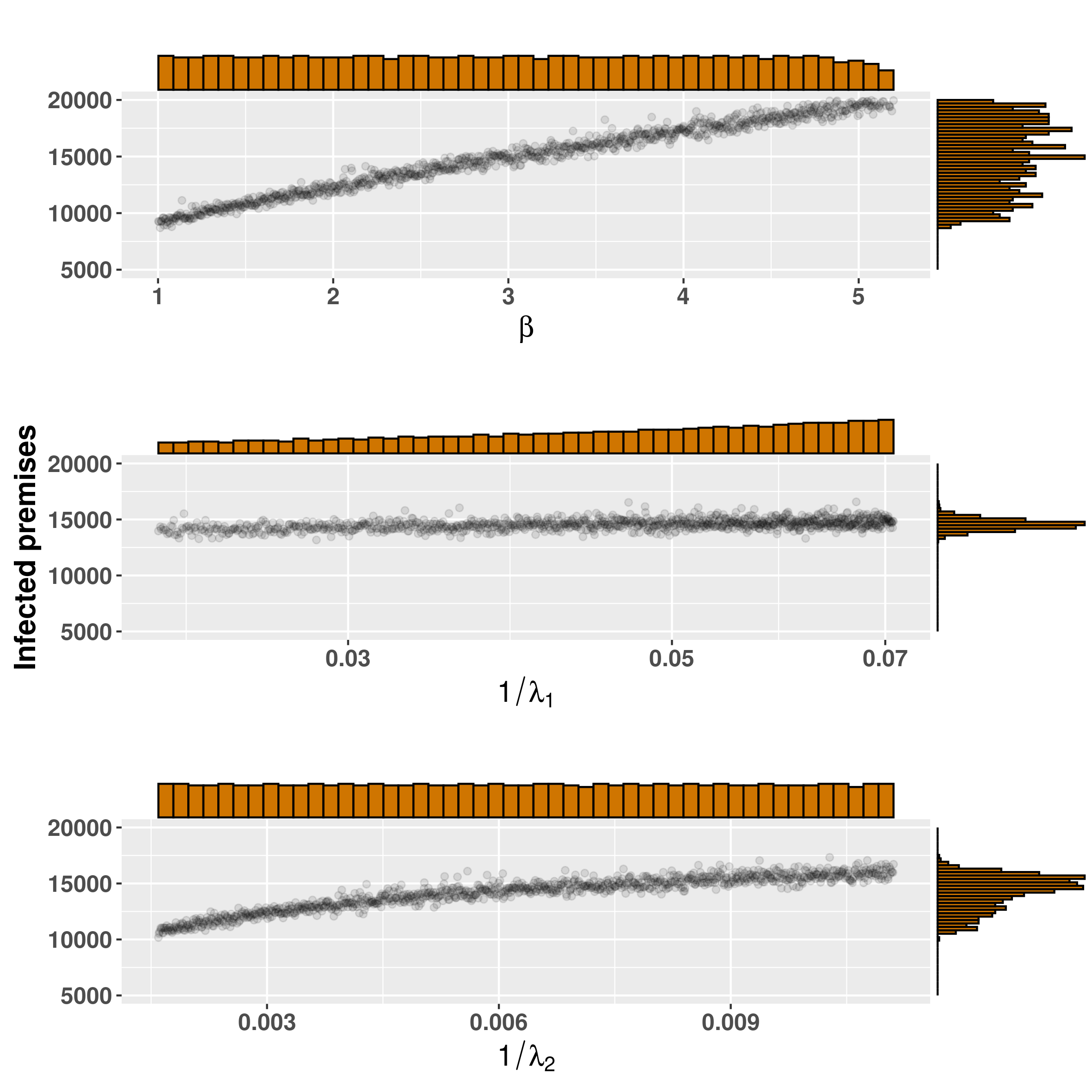

Supplement: Supplementary file 1 [file microorganisms-09-00227-s001.zip › figuras suplementar/Supplementary figure 7 sensibilidadeglobal_1000 simulations.png]

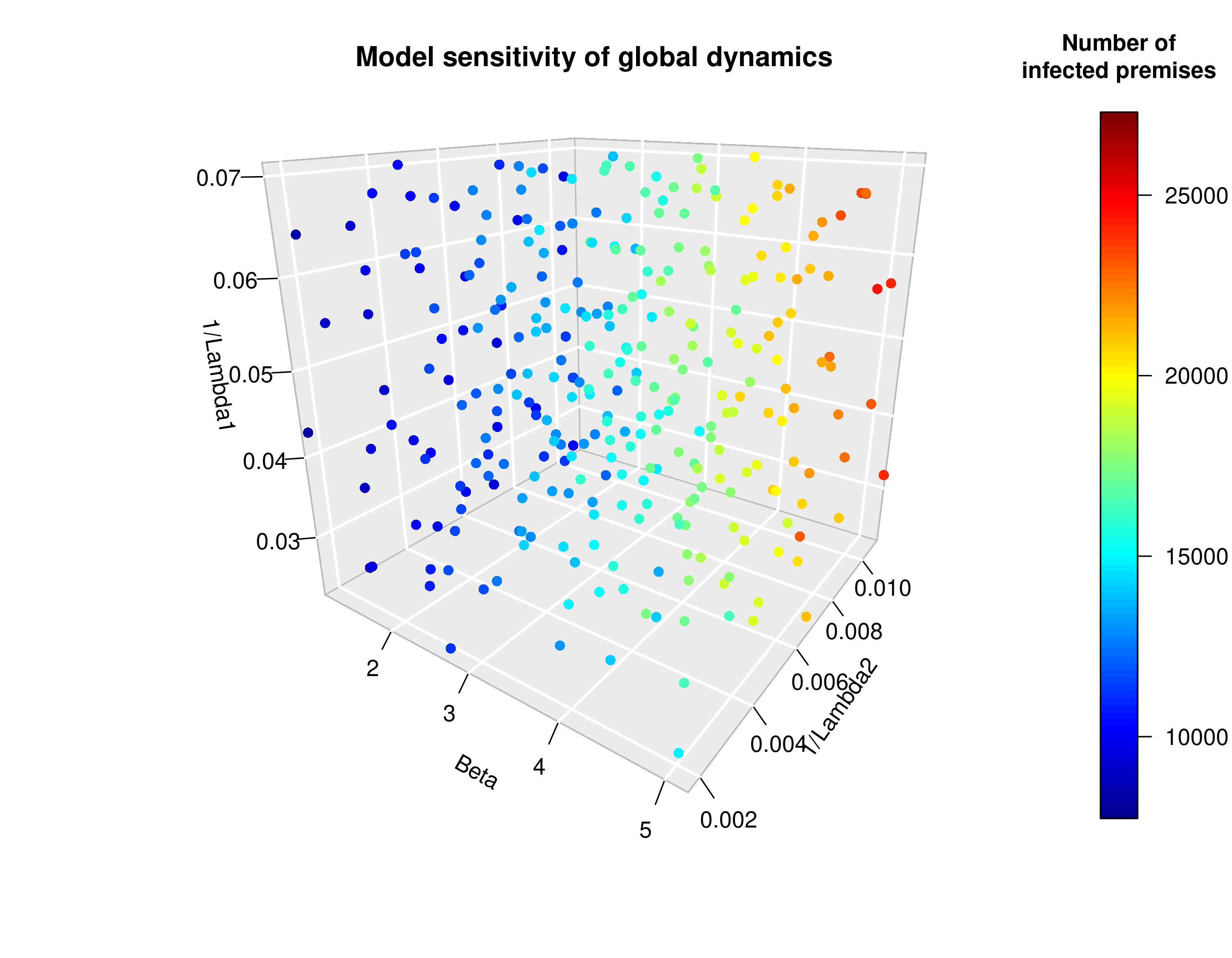

Supplement: Supplementary file 1 [file microorganisms-09-00227-s001.zip › figuras suplementar/Supplementary figure 8 3dsensiblilidade GLOBAL sori (1).png]

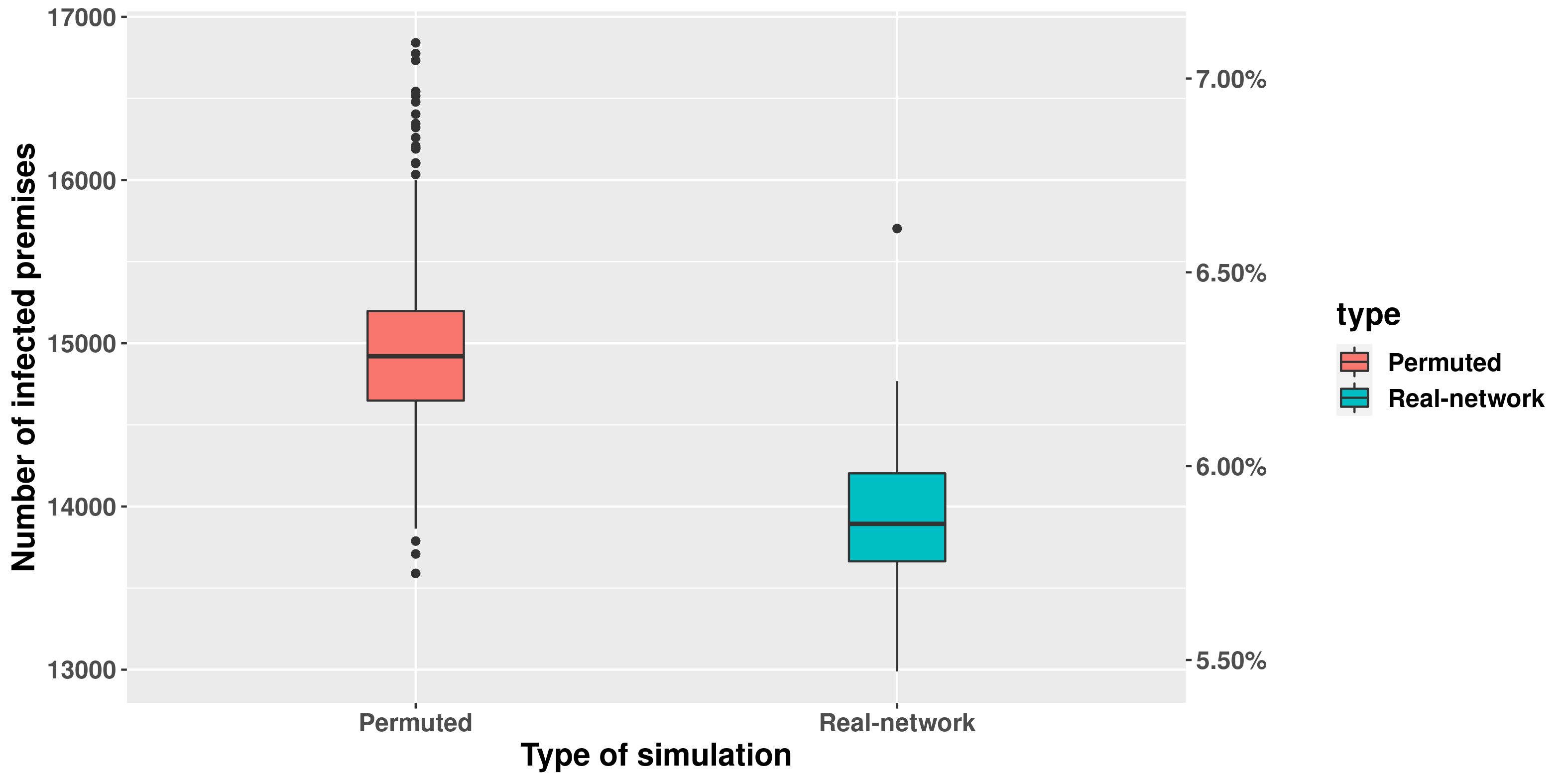

Supplement: Supplementary file 1 [file microorganisms-09-00227-s001.zip › figuras suplementar/Supplementary figure 9 plot_bocxpl.png]

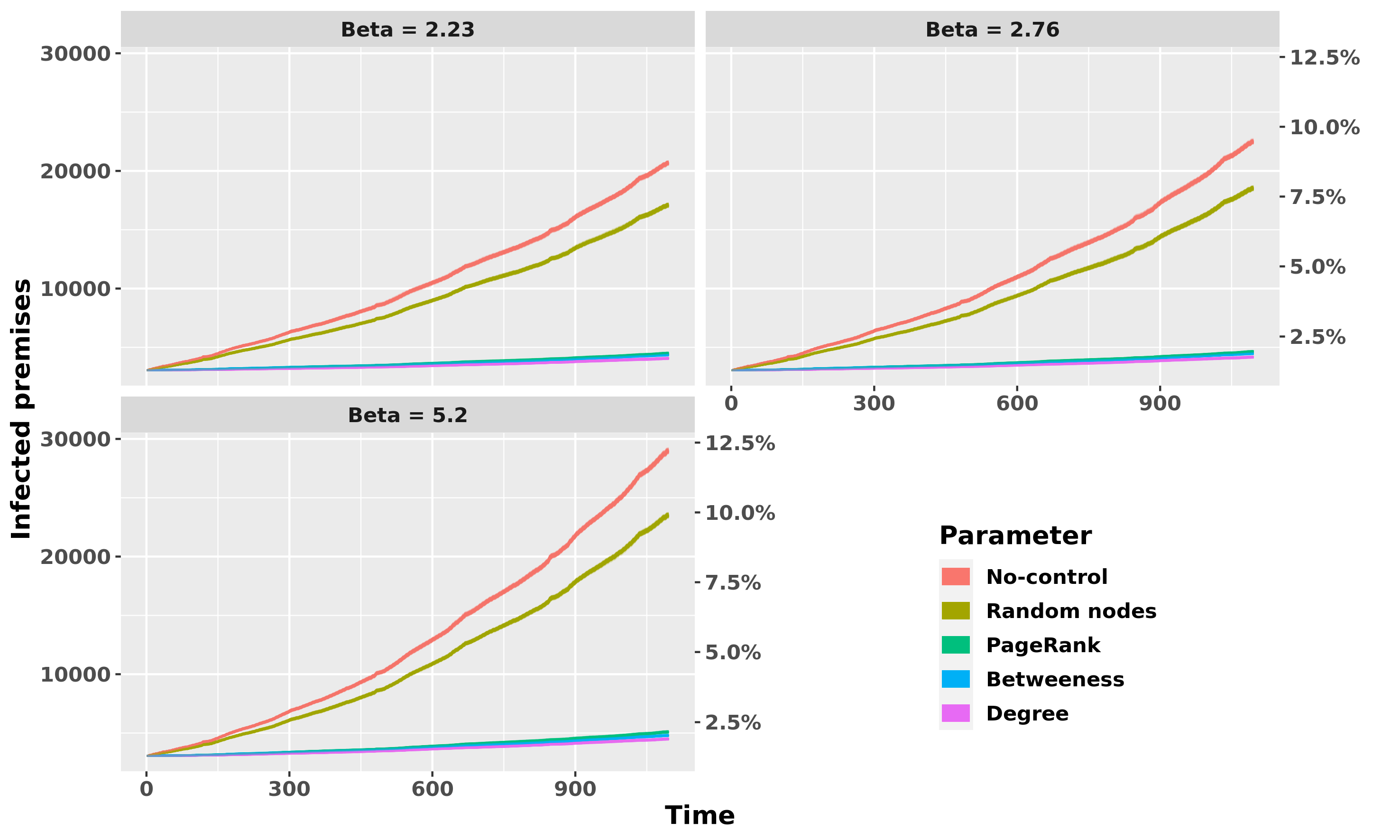

Supplement: Supplementary file 1 [file microorganisms-09-00227-s001.zip › figuras suplementar/Supplementary figure 4 TLS_sori_spread_simulation_tll2.png]

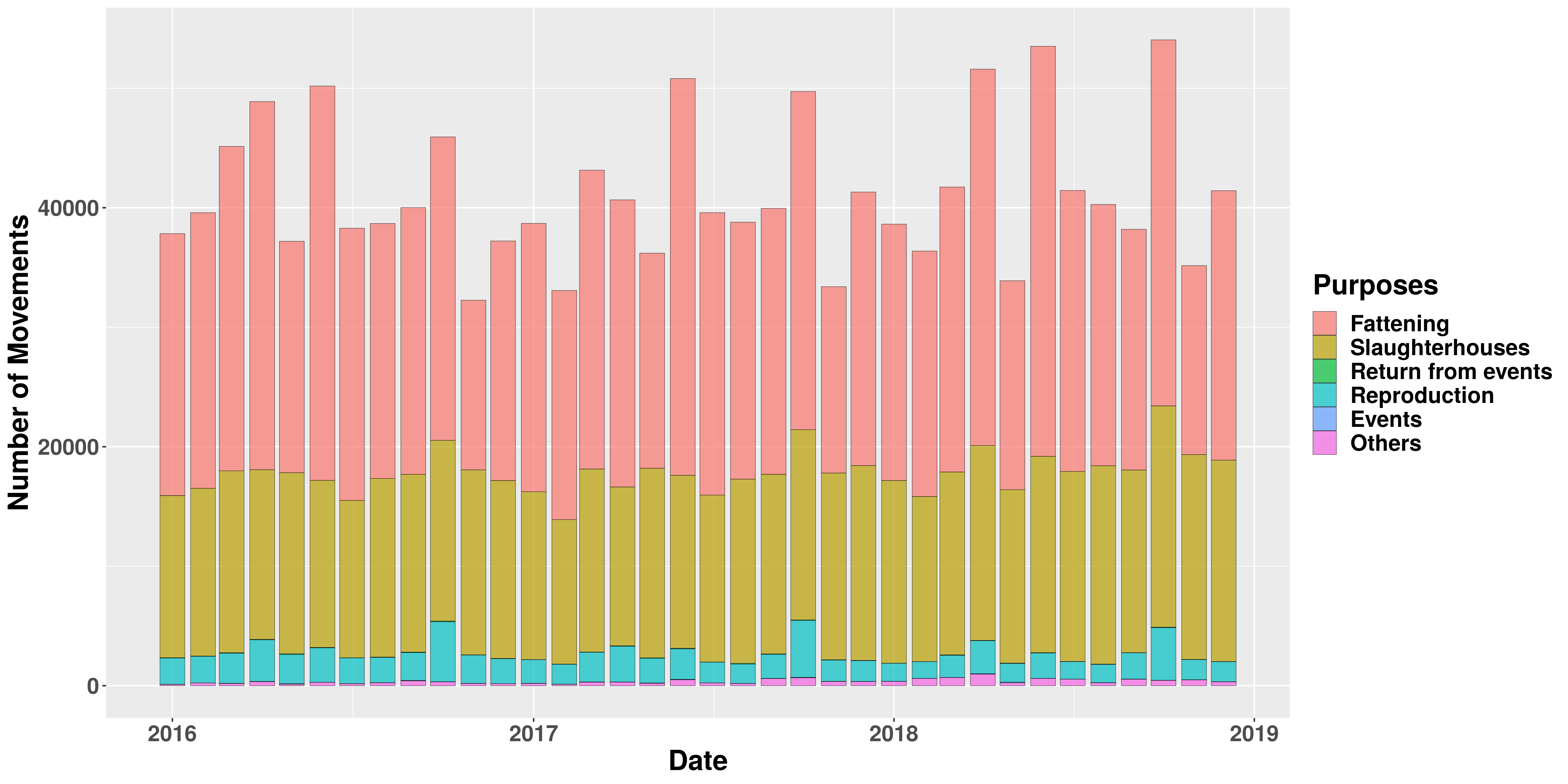

Supplement: Supplementary file 1 [file microorganisms-09-00227-s001.zip › figuras suplementar/Supplementary figure1 .png]
